# Presence of Disinfection Byproducts in Public Swimming Pools in Medellín, Colombia

**DOI:** 10.3390/ijerph17134659

**Published:** 2020-06-28

**Authors:** Paula Lara, Valentina Ramírez, Fernando Castrillón, Gustavo A. Peñuela

**Affiliations:** 1 Faculty of Engineering, Pollution Diagnostics and Control Group (GDCON), University of Antioquia, Calle 70 No. 52-21, 050010 Medellin, Colombia; pao1218@gmail.com (P.L.); valentina.ramirezz@udea.edu.co (V.R.); 2 Health Authority Entity of Medellín, La Alpujarra Administrative Center of Medellín, Calle 42 No 52-106, 050015 Medellín, Colombia; fernando.castrillon@medellin.gov.co

**Keywords:** byproducts, halogenated acetic acids, pools, trihalomethanes

## Abstract

The quality of water in swimming pools is essential to avoid risks to the health of users. Medellín has more than 1000 public swimming pools, which are supervised by the Medellín Health Authority to monitor and ensure compliance with relevant regulations. The Health Authority has financed several studies related to the quality of drinking and recreational water in Medellín in order to protect consumers and users. One such study involves the evaluation of the presence of disinfection byproducts (DBP). The best known DBPs resulting from disinfection with chlorine are trihalomethanes (THMs) and halogenated acetic acids (HAAs), as well as other minorities such as chloramines or halophenols (HPs). DBPs pose a greater risk in swimming pool water because there is a greater possibility of ingestion, since exposure occurs through several routes at the same time (direct ingestion of water, inhalation of volatile or aerosol solutes, dermal contact and absorption through skin). In the present work, high concentrations of THMs and HAAs were detected in the public swimming pools selected in the study, but the presence of HPs was not detected in the pools.

## 1. Introduction

Disinfection byproducts (DBPs) are toxic, bioaccumulative, and carcinogenic, and their formation occurs through the reaction between the disinfectant and organic compounds [1]. There are several disinfection techniques to eliminate pathogenic microorganisms, among which are ultraviolet radiation, solar disinfection, chlorine dioxide, slow filtration, and the use of chemical compounds such as hydrogen peroxide, ozone, and chlorine compounds. Chlorine compounds are the most commonly used due to their low cost, disinfecting power, residuality, biocidal properties, and easy use [2]. In swimming pool disinfection, the most commonly used in Medellín are sodium hypochlorite and calcium hypochlorite [3]. The residual free chlorine is HOCl (hypochlorous acid) and OCl^−^ (hypochlorite), being that HOCl is a much better oxidizing and disinfecting agent than OCl^−^. The Secretary of Health of Medellín established through Resolution 2018060366702 of 2018, a concentration of residual free chlorine between 1.0–5.0 mg/L for pools. Residual free chlorine generates DBPs [4], which are a group of organic and inorganic compounds formed by reactions between disinfectants and organic matter present in water [5].

Trihalomethanes (THMs) and halogenated acetic acids (HAAs) are the most commonly found DBPs in drinking water [4] and in swimming pools [6], but there are other minorities such as chloramines or halophenols. At least 600 DBPs have been identified, including halogenated acetonitriles (HANs), chloral hydrate, halophenols, haloketones, chloropicrin, chloride, and cyanogen bromide [7,8]. In swimming pool waters, greater toxicological effects were found to be associated with halo-acetonitrile (HANs) than with THMs and HAAs [9]. The formation of DBPs depends on factors such as temperature, pH, contact time, inorganic and organic compounds present in the water [10,11], type of organic matter, and concentration of the disinfectant [12]. The type of disinfectant also affects the type of DBPs. Some researchers reported that pools treated with chlorine, ozone/chlorine, and electrochemically generated mixed oxidant (EGMO), produce different total concentrations (sum of THMs, HAAs, and HANs), giving 180, 33, and 140 μg/L, respectively, for each of the three treatments [9].

DBPs pose a higher risk in swimming pools because there is a greater possibility of ingestion, and exposure occurs through several routes at the same time (direct water intake, inhalation of volatile or aerosol solutes, dermal contact, and absorption through skin) [13]. In swimming pools, bathers contribute organic matter [14], generating complex mixtures of DBPs [15] and increasing the concentration of DBPs in the water and air through the continued reaction with disinfecting agents. The highly varied anthropogenic organic load generated by bathers through sweat, urine, fecal residues, skin particles, hair, microorganisms, cosmetics, and other personal care and grooming products produces complex chemical reactions in swimming pool water, forming many DBPs [15]. It should be noted that indoor pools have up to five times more genotoxicity than outdoor pools, possibly because exposure to the open air can increase the volatilization of chemical contaminants, reducing their genotoxicity [9].

Ways to minimize the presence of DBPs include limiting the generation of organic load, for example, by encouraging bathers to take a good shower before entering pools and to not urinate while swimming, managing disinfectants to avoid the generation of DBPs, and performing efficient disinfection treatment, including frequent backwash and shock chlorination [9].

The German standard DIN 19643-1 for pools sets a maximum value of THMs at 20 µg/L of THMs [16], while France establishes a maximum of 100 µg/L for THMs [17]. In Latin America, there are no established maximum values of DBPs in public swimming pools, and studies have not been carried out on the variation of the concentration of DBPs due to the presence of bathers, water temperature, and free residual chlorine concentration.

Medellín is a city with a temperature between 24 °C and 30 °C throughout the year, with a population of more than 2,500,000 inhabitants, and more than 1000 public swimming pools. For this reason, the Health Authority Entity of Medellín applied measures to control many sources of pollution in public pools, for example, rigorously requiring that the pool be isolated from green areas, and that it has showers and a foot washing system. Despite this, the irresponsibility of users in not using the shower to eliminate body fluids, food waste, products such as sunscreen, and foot residues, cause organic matter and microorganisms to enter the pools, increasing the amount of DBPs.

## 2. Materials and Methods

### 2.1. Samplings in Selected Public Swimming Pools

The samples were taken by means of point sampling in the pools of six public establishments. One of these establishments (B) occupies 17 hectares, with several pools, facilities for the practice of different athletic disciplines, and a bike path. Another is the Medellín an aquatic complex (C), with a monthly admission of about 45,000 people, and where highly competitive swimmers from Medellín practice.

In each swimming pool, four monitoring sessions were carried out: in times of high influx of bathers, in times of low influx of bathers, on a rainy day, and on a sunny day. Sampling during periods of high and low influx of bathers was scheduled according to the holiday season or weekends.

### 2.2. Disinfection Byproducts (DBPs)

The samples were maintained at 0 °C while being transported in glass containers to the Pollution Diagnostic and Control Group—GDCON laboratory. THMs, HAAs, and HPs analyses were performed using a gas chromatograph with a micro-electron capture detector (micro-ECD). For THMs, the ASTM D6520-06 [18] method was followed, which consists of the extraction of the analytes using a fused silica fiber and subsequent reading with the equipment by thermal desorption. HAAs were analyzed according to EPA method 552.2 [19], involving a liquid–liquid extraction of 40 mL of sample with 4 mL of methyl tert-butyl ether (MTBE) and analysis with a capillary gas column (GC primary column—DB-5625). The HP analyses were performed according to the UNE-EN 12,673 [20] gas chromatographic separation method.

### 2.3. Byproducts Analyzed and Limit of Quantification (QL)

THMs (QL = 5 µg/L is the same for each compound): chloroform, bromodichloromethane, dibromochloromethane, bromoform.

HAAs (QL = 5 µg/L is the same for each compound): chloroacetic acid, bromoacetic, dichloroacetic, bromochloroacetic, trichloroacetic, dibromoacetic, bromodichloroacetic.

HPs (QL = 17 µg/L is the same for each compound): 2-chlorophenol, 4-chloro-3-methylphenol, 2,6-dichlorophenol, 2,4-dichlorophenol, 2,6-dichlorophenol, 2,4-dichlorophenol, 2,4,6-trichlorophenol, 2,3,6-trichlorophenol, 2,3,5-trichlorophenol, 2,4,5-trichlorophenol, 2,3,4-trichlorophenol, 3,4,5-trichlorophenol, 2,4,6-trichlorophenol, 2,3,6-trichlorophenol, 2,3,5-trichlorophenol, 2,4,5-trichlorophenol, 2,3,4-trichlorophenol, 3,4,5-trichlorophenol, 2,6-dibromophenol, 2,3,4,5-tetrachlorophenol, 2,3,5,6-tetrachlorophenol, 2,3,4,6-tetrachlorophenol, 2,3,4,5-tetrachlorophenol, 2,3,5,6-tetrachlorophenol, 2,3,4,6-tetrachlorophenol, 2,4,6-tribromophenol, pentachlorophenol. 

## 3. Results

### 3.1. Results of THM and HAAs Concentration

Table 1 shows the results of the THMs and HAAs; HP byproducts evaluated in the selected public pools were not detected.

Concentrations of THMs were higher in some pools, and HAAs in others. Kanan and Karanfil (2011) reported that the organic matter of pool user bodies exhibited higher formation potentials of HAAs than THMs [14].

The concentrations of THMs and HAAs were higher on rainy days than on sunny days (when temperature is around 32–34 °C), which is due to the fact that the higher temperature favors the volatilization of THMs and HAAs, decreasing their concentration in the water. It is clearly observed that, when the pool has more users, the concentration of THMs is higher than when there are fewer users. This indicates that the organic matter from users contribute to forms THMs.

Most of the pools showed higher concentrations of HAAs when there were fewer users, which is surprising given the findings of Kanan and Karanfil (2011), who showed that the organic matter of the users’ bodies has HAAS-forming potential [14].

Samples with few users were collected on a Saturday around 9 a.m., and samples with many users were collected at around 3 p.m. It is likely that, due to the temperature on the day in Medellín (32 to 34 °C, as the samples were collected on sunny days), the pools lost HAA concentration. The HAAs formed and accumulated until 6 p.m., the time the pools close, and perhaps did not evaporate, since from 5 p.m. the temperature of Medellín begins to decrease, reaching 19 °C between 9 p.m. and 7 a.m. Therefore, the next day at 9 a.m., the concentration of HAAs may be high. This is not the case with THMs as they are more easily volatilized, even at night.

It is important to mention that the water used to fill swimming pools is also for human consumption in the city of Medellín, and this water is of good quality (our group has been monitoring the quality of the water for human consumption in the city of Medellín from 2004 to date). The concentrations of DBPs in water for human consumption in Medellín are very low (Table 2) and they comply with the regulations established in Colombia. All the pools selected in this study use water from the Ayurá or Manantiales aqueducts.

**Figure 1:**
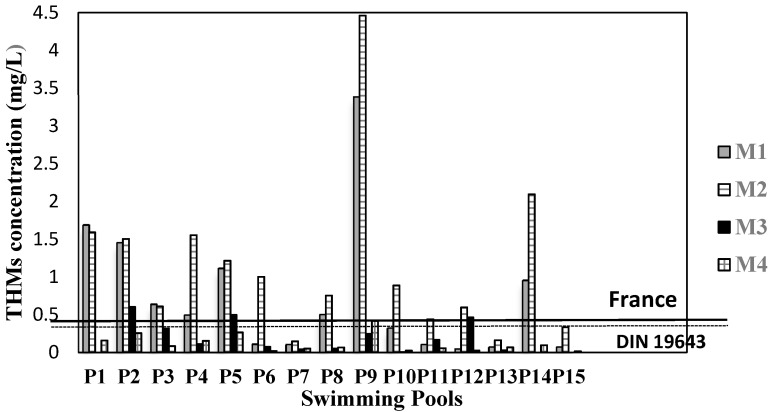
THM concentration in the public pools selected for the study. With horizontal lines is showed the regulations for the maximum value of THMs in pools of the France standard and the German DIN standard.

**Figure 2:**
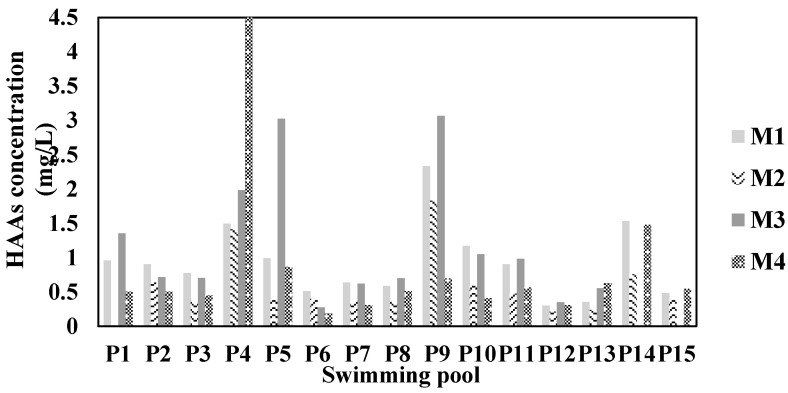
Concentration of HAAs in the public pools selected for the study.

Figure 1 and Figure 2 show how the concentration of THMs and HAAs varied in the selected pools in Medellín according to the DBP study.

The P1, P4, P5, P9, and P14 pools had the highest THMs and HAAs values. P9 corresponds to a teaching pool for babies, who generate a large amount of body fluids, favoring the production of THMs and HAAs. Eleven pools did not comply with the regulations for the maximum value of THMs in pools of the France standard and the German DIN standard (Figure 1).

### 3.2. Residual Free Chlorine Measured during the Monitoring of DBPs

**Figure 3:**
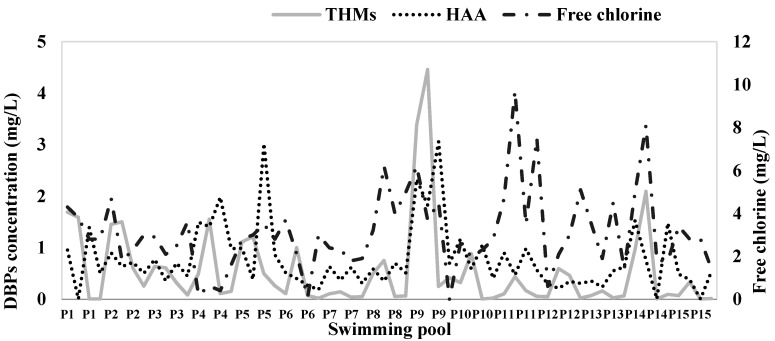
Residual free chlorine in the public pools selected for the study when sampled for the measurement of THMs and HAAs.

Figure 3 shows that the residual free chlorine measured during sampling to measure THMs and HAAs was generally well below 2 mg/L.

In general, the residual chlorine influenced the concentration of THMs and HAAs. However, the speed of formation of the DBPs depends on the pH of the water in each pool. The average pH of swimming pool water was 7.55, with a maximum value of 8.45 and a minimum of 7.05. The pKa of hypochlorous acid is 7.53 (25 °C), indicating that the majority species in some pools was hypochlorite, and in others hypochlorous acid. Hansen et al. (2013) proved that formation of THMs increases in pools when pH is increased above 7.2 [21].

### 3.3. Temperature Measured during the Monitoring of DBPs

**Figure 4:**
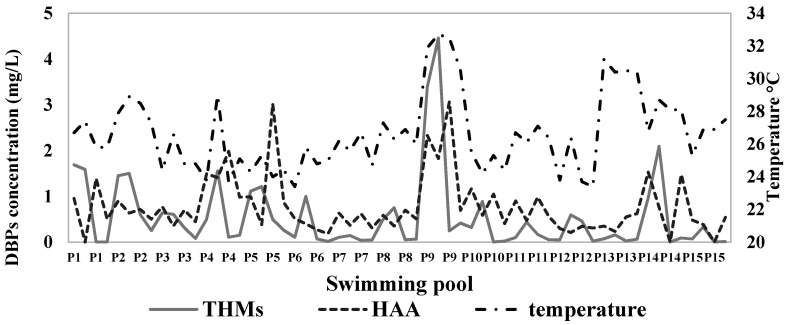
Water temperature during sampling for the measurement of THMs and HAAs in the public pools selected for the study.

Figure 4 shows water temperature during sampling for the measurement of THMs and HAAs in the public pools selected for the study. The water temperature is very influential in the concentration values of THMs and HAAs.

## 4. Discussion

To provide a comparison reference, Table 3 shows the results of THMs and HAAs in swimming pools in other countries. Based on this, THMs were found at surprisingly high values in public pools in Medellín. The values for HAAs found in public swimming pools in Medellín were also generally the highest, but they are in the range in which these byproducts were found in the United States. Despite the fact that it is mandatory for public pools in Medellín to have foot washes and showers, users often do not comply with requirements to shower thoroughly before entering the pool, which favors the organic matter of the bodies of the users or their sun protection products, which react with the chlorinated disinfecting agent to form HAAs and THMs.

Table 4 shows the maximum allowed concentrations (MAC) of THMs in swimming pool water in several European countries. Most of the samples from the public swimming pools in Medellín have levels of THMs that would not comply with the limits shown in this table. This is very worrying, because it is putting the health of bathers at risk. The Medellín Health Authority has demanded for many years that establishments apply controls on the use of swimming pools, such as keeping them isolated from green areas, and having mandatory foot washing areas and showers. Colombia does not have reference values for DBPs in swimming pools, but for drinking water it has established a maximum allowed value of 200 µg/L in THMs; there are no reference values for the other DBPs.

According to Figure 1 and Figure 2, P19 pool is highlighted (see coding in Table 1), as it had high values of THMs and HAAs in all the samplings. This pool is used for teaching babies, who because of their age are not aware of bodily functions and involuntarily urinate inside the pool and will also have products, such as sunscreen, that their parents apply to their skin. The biggest contributor to DBPs in pools is urine; it has estimated that swimming pools contain an average of 30 to 80 mL of urine for each person [36]. Therefore, there is a high input of organic matter into this pool. Urea, the main component in urine [13], was reported in swimming pools at concentrations of 0.50–2.12 mg/L [37], 0.12–3.6 mg/L [38], 0.01–0.11 mg/L [28], and 0.23 ± 0.19 mg/L [39]. Urine has other compounds such as creatinine, hippuric acid, citric acid, ammonia, uric acid, glycine, and histidine, which can produce DBPs [40]. Sunscreens released by swimmers could be the potential precursors of chlorinated and oxidized or nitrogenous DBPs [41].

DBP precursors in swimming pools waters include natural organic matter (NOM) from the filling water, body fluids from the swimmers (e.g., urine, sweat, hair, saliva, etc.), and products used by users (e.g., sunscreen, body lotion, hand soaps, laundry detergents, shampoos, hair gels) [11,29]. In Medellín, the water used to fill pools is from an aqueduct that has total maximum organic carbon values of 1 mg/L. Keuten et al. (2014) found that sweat is the body fluid that provides the second biggest contribution of organic matter to swimming pools [42]. The amount that people sweat in pools depends on both the water temperature and their level of activity. According to Keuten et al. (2014), in cold water, “you don’t sweat because the water is cooling your body down and your core temperature doesn’t rise” [42], while around 27 or 29 °C, “the cooling effect of the pool water is not enough, and your core temperature starts rising” [42]. In the selected pools of Medellín, the average water temperature was 26.64 °C, the minimum being 23.40 °C and the maximum 32.70 °C. This indicates that pools in Medellin have a significant contribution of sweat by bathers.

The results from pool P9 demonstrate that organic matter is the precursor of THMs. When there are a relatively high number of babies in the pool, the concentration values of THMs increase remarkably compared to when there are few babies in the pool. It is possible that the same occurs with HAAs; however, in the present work this was not very clear.

In some pools (Figure 3), such as P9 and P14, free residual chlorine was close to or slightly greater than 2 mg/L, and higher concentrations of THMs and HAAs were measured than in the other pools. Although in our study the relationship between residual free chlorine and DBPs was only evident in some pools, a direct correlation between free residual chlorine and DBPs has been reported in the literature [26,35,43].

Figure 4 shows that the water temperature in the pools was in the range of 23.40 °C to 32.70 °C during sampling to measure THMs and HAAs, but the pools that exceeded 28 °C, such as P4, P9, and P14, had the highest DBP values. This is in accordance with what was reported by Karanfil et al. (2015) who proved that the increase in residual free chlorine and temperature increases the concentrations of THMs and HAAs [26]. Ilyas et al. (2018) reported that total organic carbon, residual free chlorine, temperature, pH, and bromide play a fundamental role in DBP formation processes [44]. Yang et al. (2016) reported that THM concentrations almost doubled when the temperature increased from 25 to 40 °C [43]. However, the low volatility of HAAs causes their accumulation in the water, causing their concentrations to increase to a very high level over time [45], while the high volatility of THMs prevents their accumulation.

HPs were detected in waters for human consumption in concentrations between 0.010 and 0.486 µg·L^−1^ in Poland [46], between 0.008 and 0.238 µg·L^−1^ in Rio de Janeiro [47], and on average 0.06 µg·L^−1^ in Tunisia [48]. In the present study, we did not detect HPs in swimming pools or water for human consumption. There are very few reports of HPs in swimming pool waters because they are in concentrations that are too low to detect easily. By themselves, HPs are generally in low concentration in water for human consumption. During hypochlorite disinfection, HPs are formed less easily than THMs and HAAs, because hypochlorous acid does not react easily with phenolic compounds, although hypobromous acid does so slightly faster [49]. Despite this, a wide variety of HPs were reported in water for human consumption [50]. Among the few reports on the detection of HPs in swimming pool waters is that of Xiao et al. (2012), who verified the presence of 2,4-dibromophenol, 2,4-dichlorophenol, 2-bromophenol, 2,6-dibromo-4-nitrophenol, 2-bromo-6-chloro-4-nitrophenol, and 2,6-dichloro-4-nitrophenol, which were fully identified using the powerful precursor ion scan method with electrospray ionization triple quadrupole mass spectrometry [51]. They found small amounts of bromide in the chlorine sanitizing agent.

### 4.1. Statistical Analysis

#### 4.1.1. Correlations

**Figure 5:**
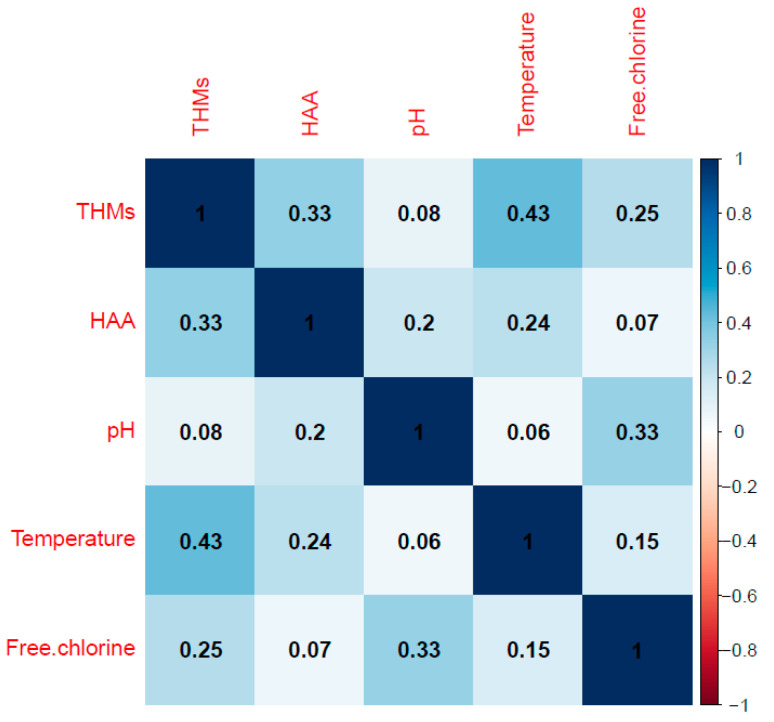
Correlation graph.

In order to determine if there are significant linear relationships between the variables, the correlation matrix was calculated (Figure 5 and Table 5). It is observed that all relationships are direct, the strongest being between temperature and THMs.

#### 4.1.2. ANOVA Analysis of Variance

In order to determine the influence of the pool on the concentration of THMs and HAAs, the analysis of variance was performed and results presented in Table 6 and Table 7. With a confidence level of 95%, lower *p*-values (to 0.05) are taken as significant. In conclusion, the HAA concentration values depend on the pool (0.003608 < 0.05), while THM concentration values do not (0.1911 > 0.05).

It should be noted that power transformation was performed on the variables to guarantee normality and the assumptions of nonautocorrelation, homoscedasticity, and normality of the residuals were validated.

## 5. Conclusions

The pool that has the most THMs and HAAs is P9. This is used for swimming lessons for babies, which indicates that more bodily waste is released into this pool, along with other products, such as sunscreens.

It was observed that the concentration of free residual free chlorine and the temperature favor a higher concentration of THMs and HAAs.

The results are consistent with what was reported previously, that the concentration of HAAs is higher than that of THMs.

The high concentrations of THMs and HAAs found in the public pools of Medellín are certainly not due to the water with which the pools are filled, which is also water for human consumption in Medellín. This is good quality water with very low concentrations of THMs and HAAs, which comply with the maximum permitted values for these compounds.

HPs were not detected, which is consistent with the very few reports confirming the presence of these compounds in swimming pool water.

We recommended that the environmental health authority carry out annual monitoring of DBPs in all the public swimming pools in Medellín, and carry out training with the managers of public swimming pools on how to minimize the presence of DBPs to avoid health risks for pool users.

## Figures and Tables

**Table 1:** Results of trihalomethanes (THMs) and halogenated acetic acids (HAAs) concentration in public swimming pools (four monitoring times: M1, M2, M3, M4). M1: a rainy day; M2: a day when there were many users in the pool; M3: a day when there were few users in the pool; M4: a sunny day.

Establishment	Pool	Code	THMs (µg/L)				HAAs (µg/L)			
			M1	M2	M3	M4	M1	M2	M3	M4
A	Pool A	P1	1691	1591	ND	158	956	ND	1352	505
	Pool B	P2	1453	1504	603	255	903	638	717	505
B	Waves	P3	638	609	317	83	776	357	703	450
	Children Round	P4	495	1553	110	152	1495	1412	1984	985
	Multiple Didacts	P5	1115	1214	497	264	991	385	3023	865
	Slides	P6	110	1000	73	18	513	401	273	187
C	Olympic	P7	106	145	40	49	636	371	621	310
	Ornamental Jump	P8	501	753	53	65	589	354	701	512
	Teaching Babies	P9	3389	4461	248	417	2332	1828	3065	696
	Adults School	P10	322	886	6	23	1170	590	1050	407
	Children School	P11	105	439	167	55	903	474	985	567
D	Adults	P12	48	595	464	24	299	212	348	310
E	Adults	P13	72	161	27	67	354	238	553	629
F	Semi-Olympic	P14	956	2094	ND	94	1531	757	ND	1480
	Waterfalls	P15	71	333	ND	13	485	385	ND	545

**Table 2:** Concentration of THMS and HAAs in drinking water from the city of Medellín, samples collected at different points in the aqueduct network by the GDCON.

Aqueduct	Concentration THMs (µg/L)		Concentration HAAs (µg/L)	
	M1	M2	M1	M2
Aqueduct La Montaña	64	7	9	14
Aqueduct Villa Hermosa	201	6	56	<5
Aqueduct Ayurá	133	6	15	6
Aqueduct Manantiales	136	20	60	44

**Table 3:** Concentrations of THMs and HAAs in swimming pool waters in other countries (Table reported by [22] and supplemented by us).

Country	THMs (µg/L) Average or Range	HAAs (µg/L) Average or Range	References
Italy	17.8–70.8		[23]
Italy	36.9–65.1	11–403	[24]
Italy	11–85		[25]
US	80 (26–213)	1541 (172–9005)	[26]
US	~25	1442 (800–2430)	[27]
US	81 (12–282)		[28]
Germany	39 (5–125)		[29]
Germany	35–47		[30]
Germany		218 (111–390)	[31]
Germany	7.1–24.8		[32]
England	132 (57–223)		[33]
Canada	38.1	257.6	[34]
Canada	29 (13–46)		[31]
France	70 (50–92)	116 (109–132)	[15]

**Table 4:** Maximum allowed concentrations (MAC) of THMs in swimming pool water in several European countries [22].

Country	MAC (μg/L)	Comments	References
Germany	20	THMs calculated as chloroform	[16]
Switzerland	30	THMs for indoor pools	[35]
Denmark	25 or 50	THMs (depending on the type of pools)	[35]
Belgium	100	Chloroform	[35]
France	100 or 20	THMs	[17]
United Kingdom	100	THMs	[35]
Finland	100	THMs	[35]

**Table 5:** Correlation matrix.

THMs	HAA	pH	Temperature	Free Chlorine
1				
0.33	1			
0.08	0.20	1		
0.43	0.24	0.06	1	
0.25	0.07	0.33	0.15	1

**Table 6:** ANOVA for THMs.

Variation Source	Square Sum	Degree of Freedom	Mean Square	F Statistic	*p*-Value
Swimming	54.973	14	3.9266	1.4034	0.1911
Residuals	125.911	45	2.7980		

**Table 7:** ANOVA for HAAs.

Variation Source	Square Sum	Degree of Freedom	Mean Square	F Statistic	*p*-Value
Swimming	3.2009	14	0.22864	2.8796	0.003608
Residuals	3.5730	45	0.07940		
